# The course of asthma during pregnancy in a recent, multicase–control study on respiratory health

**DOI:** 10.1186/s13223-018-0242-0

**Published:** 2018-04-17

**Authors:** A. Grosso, F. Locatelli, E. Gini, F. Albicini, C. Tirelli, I. Cerveri, A. G. Corsico

**Affiliations:** 1 Division of Respiratory Diseases, IRCCS “San Matteo” Hospital Foundation, University of Pavia, Vaile C. Golgi 19, 27100 Pavia, Italy; 2 Unit of Epidemiology and Medical Statistics, Department of Diagnostics and Public Health, University of Verona, Verona, Italy

**Keywords:** Asthma control, Pregnancy, Inhaled corticosteroids

## Abstract

**Background:**

Over the years it has been widely stated that approximately one-third of asthmatic women experience worsening of the disease during pregnancy. However, the literature has not been reviewed systematically and the meta-analytic reviews include old studies. This study aimed to examine whether the prevalence of worsening asthma during pregnancy is still consistent with prior estimate or it has been reduced.

**Methods:**

A detailed Clinical Questionnaire on respiratory symptoms, medical history, medication, use of services, occupation, social status, home environment and lifestyle was administered to random samples of the Italian population in the frame of the Gene Environment Interactions in Respiratory Diseases (GEIRD) study. Only clinical data belong to 2.606 subjects that completed the clinical stage of the GEIRD study, were used for the present study.

**Results:**

Out of 1.351 women, 284 self-reported asthma and 92 of them had at least one pregnancy. When we considered the asthma course during pregnancy, we found that 16 women worsened, 31 remained unchanged, 25 improved. Seven women had not the same course in the different pregnancies and 13 did not know. The starting age of ICS use almost overlaps with that of asthma onset in women with worsening asthma during pregnancy (19 years ± 1.4), unlike the other women who started to use ICS much later (30.3 years ± 12). In addition, the worsening of asthma was more frequent in women with an older age of onset of asthma (18 years ± 9 vs 13 years ± 10). Among women who completed the ACT during the clinical interview, the 50% of women who experienced worsening asthma during pregnancy (6/12) had an ACT score below 20.

**Conclusion:**

Asthma was observed to worsen during pregnancy in a percentage much lower to that generally reported in all the previous studies. There is still room in clinical practice to further reduce worsening of asthma during pregnancy by improving asthma control, with a more structured approach to asthma education and management prepregnancy.

## Background

Asthma is the most common respiratory disorder complicating pregnancy and it is associated with a range of adverse maternal and perinatal outcomes. Its prevalence among pregnant women varies among studies from 4 to 8% and appears to have increased over recent decades. Several studies have demonstrated that the use of inhaled corticosteroid (ICS) for the treatment of asthma does not affect fetal growth, and that maternal uncontrolled asthma has a greater impact on the fetus and placenta [1–3]. Even though asthma is a potentially serious medical condition and despite known risks of poorly controlled asthma during pregnancy, a large proportion of women still have a sub-optimal asthma control, principally due to concerns about surrounding risks of pharmacological agents, particularly ICS, and uncertainties regarding the effectiveness and the safety of different management strategies.

The course of asthma during pregnancy, evaluated in numerous retrospective and prospective studies over the years, has resulted to be variable. It has been stated that about one-third of asthmatic women experience worsening of the disease during pregnancy [4–7]. However, the literature has not been reviewed systematically and the meta-analytic reviews include old studies that vary in population characteristics such as asthma severity and treatment received. Many of these old studies have several methodologic inadequacy, such as low power or lack of control for confounders.

While a number of factors that may worsen asthma have been proposed in the literature, the mechanisms involved are largely undefined, and thus a woman’s asthma course during pregnancy is often unpredictable [8].

The aim of our study was to examine whether the prevalence of worsening asthma during pregnancy is still consistent with prior estimates or it has been reduced. For this purpose, we used data of our population-based Gene-Environment Interactions in Respiratory Disease (GEIRD) study.

## Methods

### Study design

GEIRD is a multicase–control study on respiratory health, involving seven Italian centres. Cases and controls were identified through a two-stage screening process in pre-existing cohorts and in new random samples from the Italian general population.

In the first stage (2007–2010), eligible subjects were administered a screening questionnaire on respiratory symptoms. In the second stage (2008–2016) all the responders with symptoms suggestive of asthma, chronic obstructive pulmonary disease (COPD) or chronic bronchitis (CB) and a random sample of subjects without respiratory symptoms or with symptoms suggestive of rhinitis, were referred to clinical centres to undergo the “phenotypization” protocol. Protocol and descriptive characteristics of the GEIRD study are available on the web site http://www.geird.org [9].

### Study population and procedures

For the present study we used clinical data belonging to 2606 subjects, who completed the stage 2 of the GEIRD study. Clinical data were obtained by means of a structured medical interview through the Clinical Questionnaire, a modification of the European Community Respiratory Health Survey (ECRHS) questionnaire (http://www.ecrhs.org) including detailed questions on respiratory symptoms, medical history, medication, use of services, occupation, social status, home environment and lifestyle. For the evaluation of regular ICS use and patient adherence to anti-asthmatic treatment the questions: “*Since the last survey have you used inhaled corticosteroids?*”, “*How old were you when you first started to use inhaled corticosteroids?*”, “*Have you used inhaled corticosteroids every year since the last survey?*”, “*If you are prescribed medicines for your breathing*, *do you normally take: A) all of the medicines? B) most of the medicines? C) some of the medicines? D) none of the medicines*” were used [10].

On the basis of the answers to the Clinical Questionnaire, a woman was considered to have asthma if she answered affirmatively to both questions: “*Have you ever had asthma?*” and ‘‘*Was this confirmed by a doctor?*’’.

The asthma course during pregnancy was evaluated with the specific question: “*What happened to your asthma during your pregnancies? A) got better B) got worse C) stayed the same D) not the same for all pregnancies E) don’t know*”.

Participants were considered to have allergic rhinitis if they answered positively to this question: “*Do you have any nasal allergies including hay fever?*”.

Atopy was assessed by skin prick tests (SPT). Individuals with at least one positive SPT were considered to be atopic. The allergens selected in all centres were *Cupressus arizonica*, *Graminacee mix Dermatophagoides pteronyssinus*, *Artemisia vulgaris*, *Dermatophagoides farina*, *Ambrosia artemisifolia*, *Alternaria tenuis*, *Parietaria Judaica*, *Dog dander*, *Corylus avellana*, *Cat hair*, *Olea europea*, *Betula verrucosa*, *Cladosporium herbarum* (ALK diagnostics, Denmark) (Mailing 1993).

In a subsample of 50 asthmatic women, we also evaluated the asthma control by means of the Asthma Control Test (ACT™). Patients assigned scores of 1–5 to each item, resulting in the following grading system: uncontrolled/partly controlled asthma with score ≤ 20; well controlled asthma with score > 20 [11]. These data refer to the time of the clinical interview and they cannot be used as an indicator of asthma control before pregnancy.

No clinical data before pregnancy are available.

### Statistical analysis

Women who answered “*D) not the same (asthma) for all pregnancies*” were excluded from the statistical analyses. The sample who answered “*E) don’t know*” was included in the group of those unchanged/improved asthma. The relationship of the main determinants of unchanged/improved asthma during pregnancy and worsened asthma during pregnancy was evaluated by Fisher’s exact test for categorical variables and by Wilcoxon–Mann–Whitney non-parametric test for continuous variables. The statistical analyses were performed using Stata 14.0 (StataCorp, College Station, TX, USA).

## Results

Out of 1351 women, 284 (mean age 44.4 ± 9) self-reported asthma and 92 of them had at least one pregnancy.

When we considered the asthma course during pregnancy, we found that 16 women worsened, 31 remained unchanged, 25 improved. Seven women had not the same course in the different pregnancies and 13 did not know.

Table 1 reports the results of the associations between considered factors and outcomes in the univariate analysis. Women who reported that the asthma was different from pregnancy to pregnancy were removed from the statistical analysis, because it was not possible to include it in either group, as there is no information on the number of pregnancies and on the course of asthma during each pregnancy. Instead, the sample who answered “don’t know” was included in the group of those “improved” or “unchanged” (N = 69). The reason is related to the fact that those who have no memory for a negative condition, most likely did not experience it. The worsening of asthma during pregnancy was significantly associated with a more regular use of ICS (p < 0.05). The starting age of ICS use almost overlaps with that of asthma onset in women with worsening asthma during pregnancy (19 years ± 1.4), unlike the other women who started to use ICS much later (30.3 years ± 12). In addition, the worsening of asthma was more frequent in women with an older age of onset of asthma (18 years ± 9 vs 13 years ± 10). Among 50 women who completed the ACT during the clinical interview, 16 (32%) showed uncontrolled/partially controlled asthma (score < 20). In particular, 50% of women who experienced worsening asthma during pregnancy (6/12) had ACT score below 20, versus 26% of those with no worsening asthma during pregnancy (“unchanged”/“improved”/“don’t know” group). The percentage of women with a good treatment adherence was found to be higher in those with worsening asthma during pregnancy than in the other group, even if the difference was not statistically significant (respectively 92 and 81%).

**Table 1:** Characteristics of women with worsened asthma and women with not worsened asthma during pregnancy

Characteristics	“Not worsened” asthmatics (n = 69)	“Worsened” asthmatics (n = 16)	p
Smoking status, n (%)			
Current smoker	13 (18.8)	3 (18.8)	1.0
Former/never smoker	56 (81.2)	13 (81.2)	
BMI category (kg/m^2^), n (%)			
< 18.5	3 (4.5)	0 (0.0)	0.710
18.5–24	44 (65.7)	12 (75.0)	
25–29	14 (2.9)	4 (25.0)	
> 30	6 (8.9)	0 (0.0)	
Asthmatic treatment adherence category, n (%)			
Null/poor	7 (19.0)	1 (7.7)	0.662
High/moderate	30 (81.4)	12 (92.3)	
Age of asthma onset (year), mean ± SD			
	13.3 ± 10.2	18.2 ± 9.5	0.09
Allergic rhinitis, n (%)			
No	14 (21.5)	3 (20.0)	0.602
Yes	51 (78.5)	12 (80.0)	
Atopy, n (%)			
No	10 (15.4)	2 (13.0)	0.602
Yes	55 (84.6)	13 (87.0)	
Starting age of anti-asthmatic drug use (year), mean ± SD			
	22.3 ± 15	19.3 ± 10.0	0.553
Starting age of ICS use (year), mean ± SD			
	30.3 ± 12	19 ± 1.4	0.2465
Regular ICS use, n (%)			
No	49 (71.0)	7 (44.0)	* 0.046*
Yes	20 (29.0)	9 (56.0)	

Significant p value is highlighted in italic (p < 0.05)

ICS, inhaled corticosteroid; BMI, body mass index; p, values compared between groups

When we considered smoking habits, BMI, presence of atopy and rhinitis, no statistically significant difference between the two groups was found in the univariate analysis.

## Discussion

The main finding in the present analysis is that asthma was observed to worsen during pregnancy in a percentage much lower that the one generally reported in all the previous studies, 18.8% (16/85) versus 30%. A meta-analytic review of 14 studies, conducted before 1990, assessing changes in the course of asthma throughout pregnancy suggested that approximately one-third of pregnant asthmatic women experience a symptomatic improvement, one-third experience a worsening, and one-third remain the same [12]. In our knowledge, at present, there are only few published data recently collected and the use of heterogeneous methods, such as the subjective nature and different definitions of asthma symptoms and control, makes the comparison with the older studies difficult.

The worsening of asthma during pregnancy represented an important problem in the management of the disease, taking into account the increased risk for preterm delivery, low birth weight preeclampsia and Cesarean delivery. Some studies indicated that over one-third of women may discontinue their asthma medications during pregnancy, many without consulting their doctors and that only half of the pregnant women with asthma used their controller drugs regularly during pregnancy [13, 14]. The undermedication of pregnant women with asthma may contribute to worsening of asthma symptoms in some women during pregnancy [15].

Over the past 20 years, there was an evolving understanding of heterogeneous airways disease, a broader evidence base, increasing interest in targeted treatment, and evidence about effective implementation approaches. Substantial advances have been made in knowledge about a wide range of new effective therapies and understanding of many important aspects of asthma care. In the past medication would not have included inhaled corticosteroids, which are a mainstay of treatment today. Inhaled corticosteroids are the treatment of choice for all levels of persistent asthma and asthma guidelines around the world strongly recommend that women continue their asthma medications during pregnancy to maintain adequate control [13].

It has been well documented that the course of asthma during pregnancy may be influenced by the various physiologic changes during pregnancy, as well as the severity of the pre-existing disease and that in general after delivery, asthma returns to the severity that was present before pregnancy [6, 7, 16, 17]. Our results indirectly confirm the influence of the severity of the disease in the asthma worsening that was present despite a more frequent regular use of ICS and a higher treatment adherence. A further confirmation comes from the result of a low ACT score in the subsample of 50 women.

Also, the age of asthma onset was higher in this group than in the other and it is well known that adult-onset asthma has a low remission rate, a worse prognosis and a poorer response to standard asthma treatment [18]; in general, asthma in adult-onset usually relates with severe types of the disease. The concomitant presence of rhinitis worsens the asthma control in these subjects, as shown by the Rhinasthma’s score. The score was on average higher in asthmatics who reported worsening of the disease (31.36 ± 25.92), although the difference with the comparison group was not significant, probably because of the small number of cases. Differently from Grzeskowiak et al. no influence of smoking habits and BMI on worsening asthma during pregnancy was found in our sample.

### Strengths and limits of the study

Strengths of our study include the use of data recently collected from more than 1300 women randomly drawn from the general population rather than from clinically selected groups, the collection of high quality data, the standardized questionnaires and protocol procedures. Nonetheless, our study has several limitations. The first weakness is the relatively limited number of cases, which also precludes the multivariate analysis on the factors associated with the worsening of asthma during pregnancy. Another important limitation is that we fully rely on self-reported data. In addition, we have only data on asthma control and on use of ICS referred to the time of the clinical interview and not those before pregnancy.

## Conclusion

The most interesting findings of this study are that: (1) the prevalence of asthma worsening during pregnancy is actually reduced compared to the past and (2) the worsening is significantly related to the severity of the disease, as indicated by the more regular use of ICS and by the presence of an ACT score below 20. There is still room in clinical practice to further reduce worsening of asthma during pregnancy by improving asthma control, with a more structured approach to asthma education and management pre-pregnancy.
